# Supplementary material for: Learning curve and surgical outcome of robotic assisted colorectal surgery with ERAS program
Source: Sci Rep. 2022 Nov 29;12:20566. doi: 10.1038/s41598-022-24665-w (PMC9709162; doi:10.1038/s41598-022-24665-w)
Supplement: Supplementary file 1 — Supplementary Information 1. [file 41598_2022_24665_MOESM1_ESM.zip › LSC Raw data-Ñ[▒K/132/H_ñw╝╨░O▒Kñσ.pdf]

正式摘要(電子簽章：000000223263815)

W52 / W52 英文標題(注意:無英文姓名-請補病人基本檔!) 【讀取】憑證

臺中榮民總醫院

出院病歷摘要

轉入號 : 轉出號 :

[redacted]

(8)流水編號	
(9)入院日期	110/08/10 (2021) 直腸外科 W52 - 162
(10)轉科日期	
(11)出院日期	110/08/20 (2021) 住院天數計 10 日

診斷

- 入院 :1. Colon Cancer, c T3 Nx M0, Stage III
  2. Hypertension
  3. Acute dementia suspected associated with inflmmation condition
  4. Acute Gouty arthritis
  5. Hyperthyroidism
  6. Bilateral inguinal hernia s/p hernioplasty on 2019/12/19.
- 出院 :1. Colon Cancer, c T3 Nx M0, Stage III,
  s/p laparoscopic transfer to open anterior resection, with suspected left ureter injury during operation s/p Lt D-J insertion on 2021/08/11
  pT3N1a(1/18)M0. pMMR, MD, AI+, PI+, TuDp+, CRM+, ExNd+, stage IIIA
  2. Hypertension
  3. Acute dementia suspected associated with inflmmation condition
  4. Acute Gouty arthritis
  5. Hyperthyroidism
  6. Bilateral inguinal hernia s/p hernioplasty on 2019/12/19.

主訴

- bloody stool for months

病史

- This 90 y/o male patient had history of :
  1. Colon Cancer, c T3 Nx M0, Stage III
  2. Hypertension
  3. Acute dementia suspected associated with inflmmation condition
  4. Acute Gouty arthritis
  5. Hyperthyroidism
  6. Bilateral inguinal hernia s/p hernioplasty on 2019/12/19.

  He had bloody stool since 07/26. Associated thin shaped stool was noted. His CEA on 110/07/29 showed 17. Colonoscopy on 2021/07/29 showed Adenocarcinoma,moderately differentiated. Abdomen CT on 2021/08/04 showed 4.1X1.4 cm mass with N1a lymph node involvement.
  He was admitted for surgery.

體檢發現

-
  - General appearance :
    - easy looking , conscious clear , without cardiopulmonary distress
    - Vital signs自動帶入date 體溫(BT) 脈搏(P) 呼吸(R) 血壓(BP) 身高(BH) 體重(BW) BMI
      20210810 36.3℃ 54次/分 18次/分 180/79mmHg 151cm 50.4kg 22.1
    - Vital signs: B.P. 180/79 mmHg , P.R. 54 /min , R.R. 18 /min , B.T. 36.3 ℃
  - HEENT :
    - Eyes: no anemic conjunctiva, no icteric sclera, normal EOM
    - ENT: no otorrhea, no rhinorrhea, no erythematous mucosa, no injected tonsil, no deformity

-
    - Neck: supple, freely movable, trachea midline,no deformity, no engorged jugular vein , no palpable thyroid or lymph node enlargement
    - Chest:
        - Inspection: Grossly normal, symmetric and fully expansion, no accessory muscle used, no operation scar, no spider angiomata, no gynecomastia
        - Palpation: no palpable mass or nodule, no subcutaneous emphysema, no tenderness
        - Ausculation: clear breathing sound
    - Heart:
        - Palpation: no thrill, no heave
        - Percussion: normal heart size
        - Ausculation: regular heart beat, normal S1, S2, no gallop, no murmur
    - Extremities: No deformity, full ranged of motion without limitation, no clubbing finger or nail cyanosis, no pitting edema, no ulcer or pigmentation, no obvious skin lesion, no varicose vein, no bone, joint or muscle tenderness

CRS CONDITION

- Karnofsky performance status: (100) Normal, no complaints, no evidence of disease
- PE of Abdomen:
- CEA: 17 ng/ml
- Imaging Studies:
- Colonoscope report: adenocarcinoma
- Figures:
    1. colon & rectum

手術日期及方法

- 手術日期及方法20210811 Laparoscopic Anterior resection and anastomosis - malignant
  20210811 Ureter, Double J insertion

住院治療經過

- After operation on 08/11, he recovered gradually. He was discharged today.

合併症

一般檢查

- 累積報告 :

DATE	TIME	ALB	TP	BIL.T	BIL.D	ALKP	AST	ALT	LDH	AMY	LIPASE	NH3	Ferritin	UA
1100811	1935	3.5		0.48	0.16		24	10						
1100817	0856	2.7		0.37				3						
1100819	0849	3.6												

DATE	TIME	WBC	RBC	HGB	HCT	MCV	PLT	BLST	NEUT	BAND	SEG	LYM	MON	EOS	BAS
1100811	1935	5220		12.4			218		73.4			19.3	6.9	0.2	0.2
1100817	0852	4480		10.4	29.8		203		73.2			17.2	8.3	1.3	0.0
1100819	0912	4270		11.5					64.3			23.9	8.7	2.6	0.5

DATE	TIME	NA	K	CL	BUN	CREAT	eGFR	CA	Mg	P	CRP	ESR	PCT	D-Dimer	LACTATE
1100811	1935	143	3.6		12	0.84									
1100817	0856	141	2.7		9	0.78	99.39	7.1							
1100819	0849	145	3.7					8.7							

特殊檢查

病理報告

- 活體組織切片病理報告　手術日期：**110/08/11**　病理號：**S1024792**

診斷醫師:陳志榮 報告日期:110/08/18
Pathologic diagnosis:
1. Sigmoid colon, open anterior resection --- Adenocarcinoma, moderately differentiated. Tumor invaded to mesocolic soft tissue and metastasized to mesocolic lymph nodes (1/18). The bilateral cut ends are free of tumor.

- 2. IMA root, dissection --- Unremarkable fibrovascular tissue.
3. Colon, cut end, distal, resection --- Tubular adenoma.
Ancillary study for diagnosis:
IHC stains for PMS2 and MSH6 are done on section G. Both markers are preserved.
Prognostic and predictive factor:
1. Tumor size: 5x3 cm.
2. Depth of invasion: Tumor invades to pericolorectal tissue.
3. Histologic grade: G2: Moderately differentiated.
4. Angiolymphatic invasion: Present.
5. Perineural invasion: Present.
6. Tumor deposits (discontinuous extramural extension): Present, specify number of deposits: 2.
7. Macroscopic tumor perforation: Not identified.
8. Surgical Margins: a) Proximal Margin: Uninvolved by invasive carcinoma, distance of tumor from margin: 9 cm. b) Distal Margin: Uninvolved by invasive carcinoma, distance of tumor from margin: 2 cm. c) Radial or Mesenteric Margin: Involved by invasive carcinoma (tumor present 1 mm from margin).
9. Lymph node status: Involved (1/18).
10. Extranodal involvement: Present.
11. Tumor regression grading S/P CCRT: No known presurgical therapy.
12. Type of polyp in which invasive carcinoma arose: Not identified.
13. Additional pathologic findings: Adenoma(s).
14. Pathological TNM Stage: pT3N1a(According to the eighth edition, American Joint Committee on Cancer Staging Guidelines for Tumors).
15. TNM descriptors: Not applicable.
Gross description:
The specimen consists of 1) a segment of colon, 16 cm in length and 3 cm in proximal cut end circumference, 3.8 cm in distal circumference, and mesocolic soft tissue. A gray tan firm tumor, 5x3 cm, is noted, causing the bowel total obstruction, 2 cm to the nearest cut end. On cross section, the tumor invades to the mesocolic soft tissue and close to the radial margin, and serosal retraction is also noted. The both cut ends are free of tumor grossly. 2) IMA root, a piece of tan yellow soft tissue, 2x1.8x0.8 cm. 3) distal cut end, a piece of tan mucosa-covered soft tissue, 1.8x1.5x0.5 cm.
Representative part for section: A) proximal cut end B) distal cut end C-G) tumor (mapping section: C-D) H-J) specimen 2-3 K-M) lymph nodes.
Note:
1. The interpretation of MMR panel and recommended studies are as below: (1) No loss of nuclear expression of MMR proteins: low probability of microsatellite instability-high (MSI-H) . (2) Loss of nuclear expression of MLH1 and PMS2: testing for methylation of the MLH1 promoter and/or mutation of BRAF is indicated (the presence of a BRAF V600E mutation and/or MLH1 methylation suggests that the tumor is sporadic and germline evaluation is probably not indicated; absence of both MLH1 methylation and of BRAF V600E mutation suggests the possibility of Lynch syndrome, and sequencing and/or large deletion/duplication testing of germline MLH1 may be indicated). (3) Loss of nuclear expression of MSH2 and MSH6: high probability of Lynch syndrome (sequencing and/or large deletion/duplication testing of germline MSH2 may be indicated, and, if negative, sequencing and/or large deletion/duplication testing of germline MSH6 may be indicated). (4) Loss of nuclear expression of MSH6 only: high probability of Lynch syndrome (sequencing and/or large deletion/duplication testing of germline MSH6 may be indicated). (5) Loss of nuclear expression of PMS2 only: high probability of Lynch syndrome (sequencing and/or large deletion/duplication testing of germline PMS2 may be indicated). (6) Please note: There are exceptions to the above IHC interpretations. These results should not be considered in isolation, and clinical correlation with genetic counseling is recommended to assess the need for germline testing.
2. This case has been peer reviewed by two doctors.
#T-59300_2 #M-81403_2 2 1105 S0000F
#T-C4560_2 #M-81406_2 0 1105 000000

細胞病理報告　手術日期：**107/01/08**　病理號：**C0700992**

- 診斷醫師:李欣倪 報告日期:107/01/11
  CYTOPATHOLOGICAL DIAGNOSIS:
  Thyroid gland, LN1, fine-needle aspiration,ThinPrep --- Benign,
  consistent with a benign follicular nodule.
  ADEQUACY:
  Satisfactory for interpretation,
  one slide fixed in 95% alcohol.
  MICROSCOPIC DESCRIPTION:
  Benign follicular cells.
  COMMENT:
  1.Clinical correlation is advised to ensure that the sample is
  representative. If clinical suspicion of malignancy persist,
  additional tissue sampling should be considered.
  2. Risk of malignancy in benign category is 0 to 3%. Based on the 2nd
  ed. The Bethesda System for Reporting
  Thyroid Cytopathology. Repeat fine-needle aspiration with ultrasound
  guidance is recommended.
  #T-96000 #M-00120 0 1001 000000

其它

出院時情況

- 15.計劃性出院

出院指示

出院計劃

主治醫師 2222H 林俊余 / 住院醫師 2340H 郭連雄

正式摘要(**電子簽章：000000223263815)**

W52 / W52

doctorRank:B
